# “Acute Myocardial Infarction in the Time of COVID-19”: A Review of Biological, Environmental, and Psychosocial Contributors

**DOI:** 10.3390/ijerph17207371

**Published:** 2020-10-09

**Authors:** Francesca Gorini, Kyriazoula Chatzianagnostou, Annamaria Mazzone, Elisa Bustaffa, Augusto Esposito, Sergio Berti, Fabrizio Bianchi, Cristina Vassalle

**Affiliations:** 1 Institute of Clinical Physiology, National Research Council, 56124 Pisa, Italy; fgorini@ifc.cnr.it (F.G.); elisa.bustaffa@ifc.cnr.it (E.B.); fabrizio.bianchi@ifc.cnr.it (F.B.); 2 Ospedale del Cuore G Pasquinucci Fondazione Toscana Gabriele Monasterio di Massa, via Aurelia Sud, 54100 Massa, Italy; zulachat@ftgm.it (K.C.); annamaria.mazzone@ftgm.it (A.M.); augustoesposito1990@gmail.com (A.E.); berti@ftgm.it (S.B.)

**Keywords:** COVID-19, acute myocardial infarction, cardiovascular risk factors, inflammation, pollution, fear

## Abstract

Coronavirus disease 2019 (COVID-19) has quickly become a worldwide health crisis. Although respiratory disease remains the main cause of morbidity and mortality in COVID patients, myocardial damage is a common finding. Many possible biological pathways may explain the relationship between COVID-19 and acute myocardial infarction (AMI). Increased immune and inflammatory responses, and procoagulant profile have characterized COVID patients. All these responses may induce endothelial dysfunction, myocardial injury, plaque instability, and AMI. Disease severity and mortality are increased by cardiovascular comorbidities. Moreover, COVID-19 has been associated with air pollution, which may also represent an AMI risk factor. Nonetheless, a significant reduction in patient admissions following containment initiatives has been observed, including for AMI. The reasons for this phenomenon are largely unknown, although a real decrease in the incidence of cardiac events seems highly improbable. Instead, patients likely may present delayed time from symptoms onset and subsequent referral to emergency departments because of fear of possible in-hospital infection, and as such, may present more complications. Here, we aim to discuss available evidence about all these factors in the complex relationship between COVID-19 and AMI, with particular focus on psychological distress and the need to increase awareness of ischemic symptoms.

## 1. Introduction

At the end of 2019, the new coronavirus SARS-CoV-2 was identified as the cause of an acute respiratory infection and cause of a worldwide pandemic. At the moment, there are many unclear issues related to the pathogenesis of the infection and the reasons underlying the extremely different clinical course, from asymptomatic to severe clinical manifestations, often carried out in a very short time period. The virus enters in several cell types, including cardiomyocytes following proteolytic cleavage of its S protein by a serine protease, and binding to the transmembrane angiotensin-converting enzyme 2 (ACE2) [1]. Moreover, whether it seems that pre-existing cardiovascular (CV) risk factors and disease may increase COVID-19 susceptibility, it has been also observed that patients with CV disease may experience more severe symptoms of infection [2]. In fact, the virus can worsen underlying CV lesions, precipitate de novo acute CV events, such as acute myocardial infarction (AMI), and induce CV chronic damage [3,4]. Thus, while the focus may be on the pulmonary system, it is important to be aware of the CV implications, which can be a significant determinant for complications and mortality associated with this virus.

Nonetheless, despite these common features and interactive factors, a significant decrease in patient admissions to intensive coronary unit (ICU) has been observed following containment measures, suggesting that other determinants may reduce the capacity to quickly manage acute patients who are simultaneously or not infected with COVID-19 [5,6,7,8].

Hence, we aim here to discuss how, besides common pathophysiological mechanisms linking COVID to CV disease and favoring acute events, other factors (e.g., fear of contagion, difficulty in contacting general practitioners, attention focused on COVID-19 patients, and a massive flow of health information and disparate viewpoints) may account for the unexpected and paradoxical decrease in AMI during lockdown, unlikely caused by a real decrease in the incidence of CV events. These reflections will help us to face a possible second COVID-19 pandemic wave or other outbreaks.

## 2. Possible Causal Links between COVID-19 Infection and AMI

### 2.1. Inflammation

COVID infection may evoke a marked immune response and “inflammatory storm” (cytokine release syndrome-CRS, with an elevation of different cytokine levels, including interleukin IL-6, -7, -22, -17, chemokine ligand 2, and tumor necrosis factor α, TNFα), found associated with disease severity and mortality [9,10]. Thus, patients with preexisting atherosclerotic lesions and chronic inflammation, then infected with COVID-19, may be at higher risk of disease severity, clinical complications such as acute coronary syndrome (ACS), and mortality, and may present conduction abnormalities, atrial fibrillation, hypotension, left ventricular dysfunction, and elevation in brain natriuretic peptide (BNP) and cardiac troponins [11,12,13]. Noteworthy, recently, some authors question the “inflammatory storm” in COVID-19 infection. In fact, if elevated IL-6 levels were found in severe COVID-19 patients, their levels resulted lower than those usually observed in (non-COVID-19) acute respiratory distress syndrome (ARDS) patients [14]. Moreover, critically ill patients with ARDS and COVID-19 infection showed lower cytokine levels (IL-6, -8, and TNFα) when compared with patients with bacterial sepsis and similar values with respect to other critically ill patients [15].

Nonetheless, in this context, it is crucial to remember that all these observations and comparisons may be limited by the use of different assays/methods, still not adequately standardized.

### 2.2. Immune Status

Uncontrolled overactivation of T cells, which may present high concentrations of cytotoxic granules, can drive injury to the immune system, similar to atherosclerosis and other CV conditions [16,17].

COVID-related inflammation also promotes a prothrombotic state (elevated D-dimer levels are common in many hospitalized COVID-19 patients) that could further increase the risk of microangiopathy in multiple organs and coronary thrombosis at sites of plaque disruption, and inhibit the action of antithrombin, the protein C system, and the tissue factor pathway [18,19,20].

### 2.3. Comorbidities

#### 2.3.1. Diabetes

Type 2 diabetes mellitus (TDM2) together with hypertension are common in COVID-19 patients, with an incidence about two times higher in ICU/severe cases than their non-ICU/severe counterparts and resulting in an elevated overall death rate [21,22].

Infection of Severe Acute Respiratory Syndrome coronavirus 2 (SARS-CoV-2) in TDM2 can trigger the release of hyperglycemic hormones (e.g., glucocorticoids and catecholamines), but also hypoglycemia episodes (<3.9 mmol observed in about 10% of TDM2/COVID-19 patients, with increased pro-inflammatory monocytes and platelet reactivity) [23,24,25].

It is still currently unknown whether hyper/hypoglycemia may alter virulence or, alternatively, if the virus interferes with insulin secretion/glycemic control or development of acute complications (e.g., ketoacidosis).

#### 2.3.2. Obesity

A retrospective cohort study, which compared patients admitted for COVID-19 pneumonia in the period between February 27th and April 5th, 2020, with patients admitted for a non-SARS-CoV-2 respiratory disease during the same period in 2019, evidenced a higher frequency of obesity among SARS-CoV-2 patients, with a correlation between disease severity and increased body mass index [26]. However, this association, sometimes heralded by the media regardless of emotional consequences on the audience, should be interpreted in a wider scenario. For example, elderly subjects may also more frequently present diabetes and hypertension or obesity, and as such, may be more susceptible to infection and to develop a more serious disease, requiring hospital admission and invasive ventilation.

#### 2.3.3. Hypertension

It is still not surely assessed if hypertension increases susceptibility to COVID-19 infection. Chinese and global data show prevalence rates of 15–40%, largely in line with the rates of high blood pressure in the general population (30%), whereas other data suggest that hypertension is present in 13.4% of subjects with non-severe disease and in 23.7% of subjects with severe disease, and tripled mortality risk [27,28]. It is important to consider that these findings may be greatly affected by the higher prevalence of hypertension in elderly, which may have worse outcomes, a more severe disease course, and higher mortality than in younger patients. Accordingly, there is no evidence of increased susceptibility of hypertensive patients for COVID-19 when the association is adjusted for age and other comorbidities [29].

#### 2.3.4. Gender-related Effects

In these associations, besides aging, gender may play a role, although sex-disaggregated data for COVID-19 in several European countries show a similar number of cases between the sexes, but more severe outcomes in older men [30]. This gender-related effect could be attributable to differences in the renin–angiotensin–aldosterone system (RAAS) system (e.g., ACE2 expression increased by testosterone and reduced by estrogens), innate recognition, and biological response to virus, and may differ according to sex hormone changes that also vary with aging [31].

### 2.4. Drug Effects in the Relationship between the Cardiovascular System and the COVID-19 Infection

Drugs currently evaluated for COVID-19 (e.g., chloroquine/hydroxychloroquine—malaria treatment; tocilizumab—autoimmune disease; ribavirin/interferon alfa—hepatitis; lopinavir/ritonavir—HIV infection) have important CV side effects and toxicities, therefore requiring caution in patients with comorbidities.

In patients treated with chloroquine/hydroxychloroquine (median of treatment duration 7 years), conduction disorders were observed as the main side effects (85%), followed by ventricular hypertrophy, hypokinesia, heart failure, pulmonary arterial hypertension, and valvular dysfunction, resulting in irreversible damage or death (13% and 30%, respectively) after drug withdrawal [32]. Interestingly, a few data suggested that these drugs are associated with significant QTc prolongation, and ventricular arrhythmias, in patients with COVID-19 [33]. However, the issues of more adverse outcomes developed after patients were treated with these drugs, whether the severity of COVID-19 infection was reduced in such patients before being infected with COVID-19, the real effectiveness and safety of these drugs, as well as the appropriate dose and duration of therapy, are all aspects which require more in-depth investigation, given the still scarce evidence and the great heterogeneity of interventions and indications [34,35].

Beta-blockers, especially metoprolol, should be administered cautiously in patients under chloroquine/hydroxychloroquine therapy, due to cytochrome-P450 isoenzyme CYP2D6 modulation and decreased heart rate [36]. Ribavirin (that binds to the active site on the virus RNA-dependent RNA polymerase) and lopinavir/ritonavir (inhibiting replication of viral RNA) may interfere with many CV drugs (e.g., warfarin, rivaroxaban and apixaban, clopidogrel, statins) [37].

Moreover, in view of actual available results, if alone it seems to retain limited value against COVID-19, its combination with interferon-α or lopinavir-ritonavir increases clinical efficacy [35]. Instead, no evidence of severe adverse events, long term survival, or quality of life has been shown using aspirin or nonsteroidal anti-inflammatory drugs in COVID-19 patients, as stated by the World Health Organization [38].

ACE-inhibitors (ACEIs) and angiotensin receptor blockers (ARBs), up-regulating ACE2, could increase virus susceptibility. However, to date, there are no clear data linking the use of these therapies with an increased risk of COVID-19 or disease severity. These drugs might even increase the lung protective function of ACE2 by reducing angiotensin II through its conversion to angiotensin [32]. Hence, as RAAS-inhibitors are a therapy cornerstone after AMI, where their withdrawal may cause clinical instability (e.g., reinfarction) in high risk patients, there is currently no justification for stopping ACEIs or ARBs in patients at COVID-19 risk [39].

The dipeptidyl peptidase 4 (DPP4) receptor appears to be another gateway for the virus, in addition to ACE2. Increased DPP4 expression and activity are associated with TDM2, obesity, and metabolic syndrome, all of which have been related to COVID-19 susceptibility and severity. For this reason, it has been hypothesized that DPP4 inhibitors, known as gliptins, which vary in their interactions with the active site of the enzyme, may have immunomodulatory and cardioprotective beneficial effects in COVID-19 management [40]. However, the impact of other TDM2 drugs on the susceptibility and outcomes of COVID-19, as well as COVID-19 therapies’ effects on glucose regulation, need to be further investigated.

### 2.5. COVID-19, Acute Myocardial Infarction, and Air Pollution

Air pollution is a complex mixture of gases (including nitrogen dioxide—NO_2_; carbon monoxide—CO; sulfur dioxide—SO2; and ozone—O3), and particulate components (PM_10_ and PM_2.5_ with aerodynamic diameter ≤10 and ≤2.5 µM, respectively), which may vary depending on the source, emission rate, and sunlight and wind conditions [41].

Short and long-term exposures to air pollutants (especially PM_2.5_, but also PM_10_ and NO_2_) have been found to be related to an increased risk of segment elevation myocardial infarction (STEMI) [42,43]. Older people are generally considered to be more susceptible to the effects of air pollution because of the gradual decline in physiological processes over time as well as the presence of underlying cardiovascular risk factors (e.g., obesity, metabolic syndrome) or pre-existing coronary artery disease, chronic lung disease, or heart failure [44,45]. Evidence of a gender-differentiated effect remains uncertain and often not statistically significant, with a few studies suggesting stronger consequences among females, while others reported a larger association for males [46,47,48].

An interesting question concerns the potential association between the transmission of SARS-CoV-2 and atmospheric pollutant levels [49]. A growing body of evidence has linked short-term exposure to PM_2.5_ with mortality for total respiratory disease [50,51], and hospitalizations due to respiratory disease and acute lower respiratory infection, including pneumonia, bronchitis, and bronchiolitis [52,53,54]. Furthermore, a significant association between daily hospital admissions and daily concentrations of ambient O_3_, CO, NO_2_, SO_2_, and PM_10_ has been recently reported [55].

Epidemiological and experimental studies have shown that air pollutants can exacerbate the susceptibility and severity of respiratory virus infections, eliciting a prolonged inflammation even in young and healthy subjects [56,57,58,59]. Positive significant associations were found between air pollution and SARS case fatality in the Chinese population during the SARS outbreak in 2002 [60], as well as between the infection rate of respiratory syncytial virus in children and PM_2.5_, PM_10_, SO_2_, NO_2_, and CO [61]. Therefore, an interesting issue is whether atmospheric aerosol is able to increase the susceptibility to COVID-19 through indirect systemic effects linked to pro-inflammation and oxidation mechanisms of the lungs, immunological dysfunction, or genotoxicity [62].

Legal threshold PM_10_ exceedances (50 μg/m^3^ per day) in the Po Valley area, situated in North Italy, and the high occurrence of COVID-19 cases have focused the attention on their possible correlation [49]. Similarly, high levels of air pollution in China are well-documented [63], and an analysis of 213 cities in China indicated positive associations of short-term exposure to PM_2.5_, PM_10_, CO, NO_2_, and O_3_ with COVID-19 confirmed cases [64]. Two cross-sectional nationwide studies conducted in the United States also reported an increase in COVID-19 mortality rate correlated to prolonged single exposure to PM_2.5_ [65] and NO_2_ [66], and to long-term exposure to NO_2_ independent of long-term PM_2.5_ and O_3_ exposure. [66]. Furthermore, according an ecological macro-scale analysis carried out in 66 administrative regions in Italy, Spain, France, and Germany, five regions located in north Italy and central Spain with the highest number of fatality cases for COVID-19 showed the highest NO_2_ concentrations [67].

Nonetheless, the role of air pollution on COVID infection diffusion and severity involves a complex chain of factors (e.g., influence of air pollutants in microorganism transport, individual sensitivity to pathogens) and consideration of confounding factors (e.g., population size and density, age distribution, comorbidities, smoking habits, gender-related differences, hospital beds, number of individuals tested, healthcare capacity, phase-of-epidemic, population mobility, sociodemographic and meteorological factors, socioeconomic status, single and multi-pollutant models, different strategies for counting COVID-19-related deaths) [68]. On the other hand, the confirmation of infection requires nucleic acid testing of swabs. Hence, what we know is the number of lab-confirmed infections of tested subjects, but this may cause errors in infection count due to lack of knowledge of the real total number of people infected with COVID-19.

Further efforts are warranted also to overcome the intrinsic limit of the ecological design (not suitable for drawing conclusions about the causal relationship), establish the causal determinants of the epidemic as well as the confounding or modifying factors, and improve the strategy of data communication, especially during a pandemic crisis that elicits stress and anxiety.

SARS-CoV-2 airborne transmission has been hypothesized; thus, the high agglomeration of air pollutants could facilitate virus diffusion [62,69]. Indeed, SARS-CoV-2 RNA was detected in outdoor PM_10_, and association with virus persistence in the atmosphere was supposed [70]. Nonetheless, SARS-CoV-2 vitality and its virulence, when adsorbed on particulate matter, are currently unknown [71,72], and the viability of aerosolized SARS-CoV-2 has been demonstrated exclusively in laboratory and indoor settings [73]. Conversely, the half-life of bioaerosol could be reduced in outdoor environments in relation to specific temperature, humidity, and ultraviolet radiation conditions [62]. In this context, the analysis of PM_10_ concentration and infections before the pandemic explosion showed that cities in Piedmont had the most severe PM_10_ pollution events but lower infection cases compared to cities in Lombardy (e.g., Brescia and Bergamo), suggesting the absence of a direct contribution due to PM_10_ transport for COVID-19 diffusion [71].

Hence, whereas evidence of a causal link between PM and respiratory and CV diseases is plausible and it is believable that long-term air pollutant exposure may affect immune response and exacerbate the conditions of chronically ill patients [59,74], whether atmospheric particulates may serve as carrier of SARS-CoV-2 is still to be demonstrated by etiological studies based on short-term exposure in small geographical areas or, preferably, on individual data [75,76]. However, as precautionary airborne transmission measures are extremely cheap and can be easily implemented (e.g., effective ventilation, germicidal ultraviolet light), they could be added without effort to the other planned precautions [77].

In any case, it will be interesting to explore if pollutants can interact with COVID-19 infection to further increase the inflammatory cascade, a main culprit in the onset of acute CV events. Of interest, a recent study proposed an exciting theory on the association between pollution, COVID-19, and its impact on the high rate of infection and mortality, plausibly higher in more susceptible patients presenting pre-existing CV disease [78]. Since chronic exposure to PM_2.5_ in mice causes up-regulation of pulmonary ACE expression and activity, in patients exposed to pollutants, viral entry could be facilitated and increased viral load could result in depletion of ACE-2 receptors (through binding of SARS-CoV-2 Spike protein to ACE2) and impairment of host defenses [79].

Moreover, in experimental models, NO_2_ exposure renders animals prone to cytomegalovirus infection with a viral load 100-fold lower than in control mice and re-infection from viral sources more common, effects that add up to the increase in oxidative stress and inflammation, and reduction in macrophage function and NO_2_-related adaptive immune responses [79]. If similar effects may occur also in COVID-19 patients remains to be clarified.

Therefore, this “dangerous liaison” between some pollutants and COVID-19 might conceivably affect transmission, number of patients, severity of presentation, and number of deaths. In particular, as both factors favor myocardial injury and trigger acute myocardial events, their interaction may increase CV risk, especially in more vulnerable groups of patients, such as those with pre-existing atherosclerosis (Table 1).

## 3. COVID-related Fear and Distress

Fear, defined as “an unpleasant emotion or thought that you have when you are frightened or worried by something dangerous, painful, or bad that is happening or might happen” (Cambridge Dictionary), involves biological adaptive responses motivating a range of positive behaviors aimed at reducing the risk (e.g., social distancing, hand hygiene), if not chronic or out of proportion. In fact, the possible prospect of getting sick, the prolonged isolation and adverse economic effects, the personal and family infection fear, the uncertainty of future and crisis duration, and the overload of (mis)information may generate negative and harmful fear [98,99,100]. A study conducted in a large Chinese general population detected an elevated stress level, anxiety, and depression (8.1%, 28.8%, and 16.5%, respectively) during the COVID outbreak onset, and remained unchanged at the epidemic peak, four weeks later [101]. Similarly, approximately 25% of 7143 Chinese students experienced anxiety during the COVID-19 epidemic [102]. This symptom may be even increased in subjects with CV disease and comorbidities, where mood alterations and/or lockdown may worsen lifestyle habits and cause poor therapy adherence [103].

In addition, healthcare professionals may develop distress after facing stressful emergencies, due to the risk of infection, overwork, isolation, and fewer family contacts that may negatively affect their attention and decision making ability, indirectly worsening patient care [104,105,106].

Psychometric tools have been developed and validated to evaluate COVID-19 fear [107,108]. The COVID-19 Peritraumatic Distress Index is a self-report questionnaire that investigates anxiety, depression, specific phobias, cognitive change, compulsive behavior, physical symptoms, and social context [6]. Data obtained in 52,730 subjects using this tool evidenced that nearly 35% of the Chinese population suffered from psychological distress, in particular female participants [6].

The Fear of COVID-19 Scale (FCV-19S) is obtained by a questionnaire of seven items (total score ranges between 7 and 35, a higher sum score indicating a higher COVID-19 fear), validated and applied in different general populations (both Asian and European), which highlighted significant associations of fear with stress, anxiety, and depression [99,109,110,111]. The presence of chronic disease is related to COVID-19 fear, and females have significantly higher fear rates than males [112].

Nevertheless, these questionnaires have not yet been tested in CV patients. We administered the FCV-19S questionnaire in 30 CV outpatients and compared these results with those published relating to the general Italian population [111]. Preliminary results, which must certainly be confirmed in a larger sample, suggested higher scores in CV risk patients for both emotional (item 4) and symptomatic fear expression (items 3 and 6) (Table 2).

In particular, AMI patients may underestimate symptoms and not promptly refer to hospital, vanquishing recommended strategies based on intervention responsiveness and incurring complications due to an evolving AMI.

## 4. AMI during COVID pandemic: Fall in Admission and Delayed Access to Hospital Care

**Figure 1:**
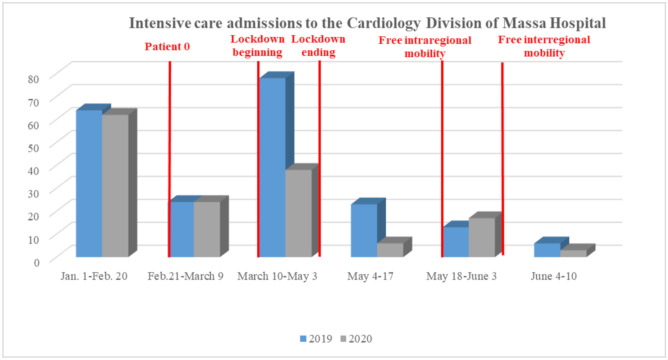
Comparison between 1 January–10 June 2019 versus 2020 segment elevation myocardial infarction admissions to the Ospedale del Cuore-Massa.

Healthcare practitioners all over the world have noticed a significant “AMI fall” during the COVID period. The number of emergency department visits in two major northern Italy referral hospitals (21 February–6 April) showed an inverse trend with daily COVID-19 mortality [113]. In Austria, a reduction of 40% in AMI admission was observed during March 2020 [114]. Data collected in the period January–March 2020 from nine high-volume USA centers, evidenced a 40% fall in the number of cardiac STEMI catheterizations [115]. The decrease was significant for STEMI (26.5%) and NSTEMI (65.1%), both in North Italy and in Central/South Italy [116]. Moreover, in a single large center in northern Italy, data obtained in March 2020 compared to March 2019 showed a significant reduction of 30% for STEMI, 66% for NSTEMI, and 50% for severe bradyarrhythmia [5]. These findings were confirmed by our experience, as we assessed a significant decline in STEMI admissions to the ICU-Cardiology Department of Ospedale del Cuore-Massa between 1 January and 10 June 2020, with respect to data collected in the same period in 2019 (Figure 1, panel A). Notably, in relation to fear, no patient with COVID-19 lab-confirmed infection was found between those admitted to our hospital, all swab-tested, until 10 June 2020.

These data are worrying considering the result obtained in a small number of Chinese AMI patients (*n =* 7), which showed a great delay in the “symptom onset to first medical contact” time after control measure implementation, when compared to 2018–2019 (5 h versus an hour and a half) [117].

Table 3 shows key time points in STEMI care in the COVID period compared to pre-/post- outbreak periods (Ospedale del Cuore-Massa). Additionally, in our experience, the major difference was in the time from “symptom onset to first medical contact”.

## 5. Discussion

The focus on the COVID-19 pandemic, which has significantly tested the health care system globally, has let the guard down against psychological effects in the general population and people with chronic diseases.

**Figure 2:**
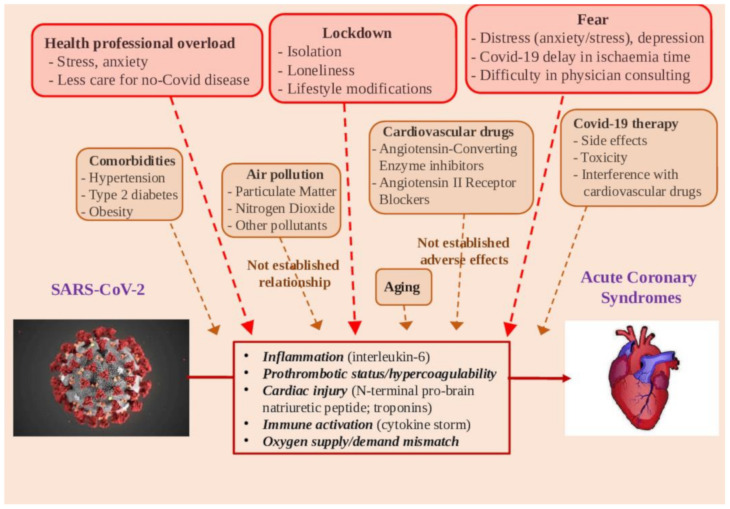
Potential determinants in the relationships between SARS-CoV-2 infection and acute myocardial infarction.

The heart–brain axis shows close interaction, as depression and anxiety are related to a higher risk of CV events and mortality [118,119,120,121,122]. Nevertheless, in this COVID-19 period, psychological load does not seem associated with CV disease exacerbation, but rather with a fall in hospital admissions. In particular, incorrect communication may have generated the fear of possible in-hospital contamination, avoiding regular checks, delaying the diagnosis of acute events, and referral to ICU units (Figure 2).

Global measures and media–health communication may have generated fear of possible in-hospital contamination, avoiding regular checks by doctors, whereas consulting cardiologists and regular drug intake can become difficult, delaying acute event diagnosis and worsening acute CVD consequences, and causing subsequent delay in referral to an integrated critical care unit. Health workers, which are potentially exposed to the pathogen and highly stressed, did not receive mental health assistance during the pandemic, and this may indirectly affect care quality (Figure 2) [123]. Furthermore, patients may suffer a lack of attention because contact with primary care professionals might be difficult due to reduction in non-urgent activity. Accordingly, it has been observed that non-COVID-19 hospital admissions significantly decrease during the outbreak, likely due, almost in part, to changes in health care decisions and/or delays in hospital access [124]. Additionally, out-of-hospital deaths could be increased, in numbers that are very complex to quantify, in terms of cardiac arrests, unexplained deaths, heart failure, and other non-COVID clinical causes, beyond the cardiovascular one [125]. Health communication is a critical tool to handle uncertainty and fear, reduce risky behavior, as well as encourage people to overcome the crisis [126]. Instead, inaccurate or unambiguous information can increase distress and elicit harmful social reactions, such as discrimination, anger, and aggressive behaviors [127]. The information about the putative relationship between environmental pollution and COVID infection is an emblematic example, which may attract immediate attention towards a recognized “enemy”, willingly identified as the co-culprit of the outbreak. In this case, the risks of oversimplification by inaccurate information—including the pitfall of meaningless correlation—should be taken into account [68].

In this scenario, the cardiology community should attempt every effort to reduce possible “collateral” damage through multiple actions:

- Attention to vulnerable subjects (e.g., elderly, frail people, patients at high CV risk);
- Correct information to patients on the delayed hospital access risks;
- Epidemiological monitoring;
- Strategies aimed to reduce distress;
- Workload for healthcare professionals based on health specialty;
- Multidisciplinary team including intensive care specialists, laboratorists, psychologists, and cardiologists;
- Teleconsultations and telemonitoring to monitor high-risk patients;
- Electronic devices/apps to help patients in their personal disease management;
- Warning receipt in case of alarming data;
- Regular, clear, and reliable information on pandemic to patients.

## 6. Conclusions

The relationship between COVID-19 and AMI is supported by many clues (Figure 2). An increased risk of AMI is likely related to COVID-19 infection, due to the inflammatory response and hypercoagulability. Accordingly, abnormalities of cardiac troponins are the most common finding in COVID-19-affected patients. Patients with pre-existing CV disease and CV comorbidities may exhibit higher vulnerability to COVID-19 and a worse clinical outcome.

The relationship of air pollution with COVID-19 needs to be established, and together with an adequate collection of health data, environmental and demographic information are crucial for studying possible associations between exposure to atmospheric pollutants, diffusion, and severity of COVID-19. Importantly, although PM and nitrogen oxides are recognized as exacerbating risk factors for ACS, their levels were reduced due to the lockdown. In northern Italy, these decreases reached values of up to 58% and 38%, respectively, for nitric oxide and NO2, whereas PM10 and PM2.5 showed a smaller decrease since they are affected by secondary emissions even from long distances [127]. While it is plausible that the observed drop in concentrations of air pollutants may have contributed to a reduction in hospital admissions for AMI, this hypothesis, and the risk quantification, remains to be demonstrated by etiological design studies based on short-term exposure assessment.

Moreover, therapies under investigation for COVID-19 infection can have significant CV side effects.

However, at this point, it is particularly important to assess the role of psychological issues, such as distress and fear. In particular, it will be interesting to understand whether a patient’s fear may reduce AMI presentation, provoking a delay in appropriate and timely revascularization in the short-term, as well as long-term increased morbidity and mortality. Moreover, it is always possible that other (also actually unknown) reasons may affect the decrease in the incidence of AMI during the lockdown. As an example, it was recently hypothesized that increase in sleep duration in the time of COVID may positively impact overall health and beneficially contribute to the observed AMI reduction [128].

In this context, every effort must be directed to clear and reliable information for general audience patients, avoiding the spread of inconsistent or distorted news that can generate fear or false optimism. As the pandemic continues, public campaigns to raise awareness of ischemic symptoms should be reinforced, as the indirect effects of the COVID-19 pandemic on non-COVID diseases can be even more catastrophic than the infection itself.

## Figures and Tables

**Table 1:** Possible mechanisms by which air pollutants and SARS-CoV-2 may trigger myocardial damage and acute myocardial events.

Air Pollution	SARS-CoV-2
Inflammatory response: increased levels of C-reactive [80], fibrinogen [81], and cytokines [82].	Severe systemic inflammation, *cytokine storm* [83].
Autonomic nervous system disruption [84]: heart rate variability decrease [80], heart rate increase [85].	Myocardial injury (elevated troponins); binding of SARS-CoV-2 to ACE2 antiviral drugs, corticosteroids, and other therapies [86].
Enhanced thrombosis/coagulation [87], fibrinolytic capacity inhibition [88].	Hypercoagulability, prothrombotic risk [89].
Oxidative stress, telomere erosion [90].	Myocardial oxygen demand supply mismatch: increased cardiometabolic demand required with the systemic infection and hypoxia caused by acute respiratory failure [13].
Vasoconstrictor increase (e.g., endothelin) [91,92].	Left ventricular dysfunction, heart failure, arrhythmias [93,94].
Atherosclerosis progression of and increased plaque rupture vulnerability [95].	Increased susceptibility to plaque rupture [13].
Oxygen saturation reduction [96].	Endothelial dysfunction, oxidative stress [97].

**Table 2:** Mean of the items of the Italian Fear of COVID-19 test, in a general population and in cardiovascular outpatients.

	General Population (*n =* 294) [106]	CV Outpatients (*n =* 45)
**FACTOR 1—Emotional Fear Reactions**		
1. I am most afraid of the coronavirus.	3.4	3.5
2. It makes me uncomfortable to think about the coronavirus	2.9	3.2
4. I am afraid of losing my life because of the coronavirus	2.4	2.9
5. When watching news and stories about the coronavirus on social media, I become nervous or anxious	2.9	3.0
**FACTOR 2—Symptomatic Expression of Fear**		
3. My hands become clammy when I think about the coronavirus	1.5	2.1
6. I cannot sleep because I’m worrying about getting the coronavirus	1.6	2.2
7. My heart races or palpitates when I think about getting the coronavirus	2.1	2.4

**Table 3:** Key time points (in minutes) in STEMI care (Ospedale del Cuore-Massa) before and after COVID-19 outbreak.

	1 January– 21 February	22 February– 3 June	4 June– 10 June
**Symptom Onset to First Medical Contact**	110 (15–570)	133 (15–600)	208 (15–1280)
**Door to Hospital Arrival Time**	95 (25–405)	94 (20–390)	83 (20–390)
**Hospital Arrival to Insufflation Time**	46 (15–120)	38 (15–90)	48 (15–120)
